# Early Effects of Aβ Oligomers on Dendritic Spine Dynamics and Arborization in Hippocampal Neurons

**DOI:** 10.3389/fnsyn.2020.00002

**Published:** 2020-02-12

**Authors:** Carolina Ortiz-Sanz, Adhara Gaminde-Blasco, Jorge Valero, Lidia Bakota, Roland Brandt, José L. Zugaza, Carlos Matute, Elena Alberdi

**Affiliations:** ^1^Department of Neuroscience, University of Basque Country (UPV/EHU) and CIBERNED, Leioa, Spain; ^2^Achucarro Basque Center for Neuroscience, Leioa, Spain; ^3^IKERBASQUE Basque Foundation for Science, Bilbao, Spain; ^4^Department of Neurobiology, University of Osnabrück, Osnabrück, Germany; ^5^Center for Cellular Nanoanalytics, University of Osnabrück, Osnabrück, Germany; ^6^Institute of Cognitive Science, University of Osnabrück, Osnabrück, Germany; ^7^Department of Genetics, Physical Anthropology and Animal Physiology, UPV/EHU, Leioa, Spain

**Keywords:** Aβ oligomers, spines, dendrites, Alzheimer’s disease, integrin β1, CaMKII

## Abstract

Alzheimer’s disease (AD) is a neurodegenerative disorder that leads to impaired memory and cognitive deficits. Spine loss as well as changes in spine morphology correlates with cognitive impairment in this neurological disorder. Many studies in animal models and *ex vivo* cultures indicate that amyloid β-peptide (Aβ) oligomers induce synaptic damage early during the progression of the disease. Here, in order to determine the events that initiate synaptic alterations, we acutely applied oligomeric Aβ to primary hippocampal neurons and an *ex vivo* model of organotypic hippocampal cultures from a mouse after targeted expression of EGFP to allow high-resolution imaging and algorithm-based evaluation of spine changes. Dendritic spines were classified as thin, stubby or mushroom, based on morphology. *In vivo*, time-lapse imaging showed that the three spine types were relatively stable, although their stability significantly decreased after treatment with Aβ oligomers. Unexpectedly, we observed that the density of total dendritic spines increased in organotypic hippocampal slices treated with Aβ compared to control cultures. Specifically, the fraction of stubby spines significantly increased, while mushroom and thin spines remained unaltered. Pharmacological tools revealed that acute Aβ oligomers induced spine changes through mechanisms involving CaMKII and integrin β1 activities. Additionally, analysis of dendritic complexity based on a 3D reconstruction of the whole neuron morphology showed an increase in the apical dendrite length and branching points in CA1 organotypic hippocampal slices treated with Aβ. In contrast to spines, the morphological changes were affected by integrin β1 but not by CaMKII inhibition. Altogether, these data indicate that the Aβ oligomers exhibit early dual effects by acutely enhancing dendritic complexity and spine density.

## Introduction

Alzheimer’s disease (AD) is the most prevalent neurodegenerative disorder in the elderly. The progressive increase in the amount of soluble and insoluble amyloid-β (Aβ) peptide in the brains of AD patients is thought to initiate the disease (Glenner and Wong, 1984). The consequences of Aβ accumulation are multifold and include synapse deterioration and loss, inflammation and ultimately, cell death (Cline et al., 2018).

Loss of dendritic spines and changes in their morphology correlate with alterations of synaptic networks and the extent of cognitive decline in AD patients. Changes in dendritic spines occur in the early AD stages, prior to neuronal death and amyloid plaque formation (DeKosky and Scheff, 1990; Terry et al., 1991). Dendritic spines can be classified into three specific categories based on their overall morphology: stubby, mushroom and thin (Kasai et al., 2003; Hayashi and Majewska, 2005; Bourne and Harris, 2008). Relatively stable mushroom spines are large, mature spines and they form strong synaptic connections and supposedly may represent sites of memory storage. In contrast, thin and stubby represent immature and more dynamic spines. Thin spines are proposed to be “learning spines” responsible for the formation of new memories. Stubby spines could represent general precursors from which thin or mushroom spines protrude (Hayashi and Majewska, 2005; Petrak et al., 2005; Bourne and Harris, 2007) or may form as a result of mushroom spine elimination (Hering and Sheng, 2001). Indeed, a shift from mushroom to stubby spines was observed in cortical biopsies from AD patients (Androuin et al., 2018). Moreover, a reduction in the mushroom spine density occurs in AD mouse models (Saito et al., 2014; Sun et al., 2014), in *ex vivo* hippocampal slice cultures from AD transgenic mice (Tackenberg and Brandt, 2009; Penazzi et al., 2016), and under conditions of Aβ toxicity *in vivo* and *in vitro* (Popugaeva et al., 2015; Qu et al., 2017). Additionally, recent findings suggest that dendritic spine plasticity can provide cognitive resilience against dementia among the elderly with AD pathology (Boros et al., 2017).

*In vivo* studies in AD mouse models revealed that Aβ deposits have a direct toxic effect on neurites, including dendritic simplification, loss of dendritic spines, and neuritic dystrophies (Spires et al., 2005; Meyer-Luehmann et al., 2008). In addition, a CA1-specific dendritic simplification is induced by Aβ and involves dysregulation of microtubule dynamics by dendritic tau, which becomes dephosphorylated at certain sites; dendritic simplification is mechanistically distinct from spine change and neuron loss (Golovyashkina et al., 2015). However, it is unknown, which are the early events that initiate the Aβ-induced dendritic simplification.

An open question for understanding AD pathology is how soluble Aβ contributes to dendritic spine loss and dendritic simplification in early disease stages. There are a large number of putative Aβ receptors (Jarosz-Griffiths et al., 2016), however, their impact on dendritic spine dynamics is still unresolved. Integrins are a large family of extracellular matrix receptors. They are present in excitatory synapse post-synaptic densities and modulate responses including the formation and stabilization of dendrites and dendritic spines (Kerrisk and Koleske, 2013; Park and Goda, 2016). In fact, forebrain-specific knockdown of *Itgb1* (encoding β1-integrin) results in dendrite retraction in hippocampal CA1 starting during late postnatal development in mice (Warren et al., 2012).

Here, we have examined acute effects of soluble Aβ_42_ on spine dynamics, dendritic alteration, and signaling pathways. We employed *in vitro* and *ex vivo* model of hippocampal neurons after targeted expression of EGFP to allow high-resolution imaging followed by algorithm-based evaluation of spine changes and alterations of dendritic arborization. Our results indicate that spine stability and dynamics are modulated by oligomeric forms of Aβ peptide. We also found that acute Aβ oligomers promote an increase in spine density by mechanisms involving integrin β1 and CaMKII signaling. Moreover, Aβ promoted dendritic complexity in CA1 hippocampal neurons, and this effect is mechanistically distinct from spine changes.

## Materials and Methods

### Primary Hippocampal Neuron Culture

Hippocampi were dissected from the brains of E18 Sprague-Dawley rat embryos according to previously described procedures with minor modifications (Baleriola et al., 2014). All experiments were conducted under the supervision and with the approval of the Animals Ethics and Welfare Committee of the University of the Basque Country in accordance with the Directives of the European Union on animal ethics and welfare. All possible efforts were made to minimize animal suffering and the number of animals used. Hippocampi were subsequently incubated at 37°C and washed in Hank’s balanced salt solution and resuspended in plating medium (10% fetal bovine serum, 2 mM L-glutamine, 50 U/ml penicillin-streptomycin, 1 mM sodium pyruvate in Neurobasal). Then, hippocampi were dissociated mechanically with a pipette followed by a flame-polished Pasteur pipette. After dissociation, cells were passed through a 40 μm cell strainer (VWR, Radnor, PA, USA) and centrifuged at 800 rpm for 5 min at 4°C.

Cells were resuspended in complete medium to a final concentration of 2 × 10^5^ cells in 24-well plates and seeded onto poly-L-ornithine-coated glass-bottom μ-dishes (Ibidi GmbH, Gräfelfing, Germany). On DIV 1, culture medium was replaced with growth medium (B-27 supplement, 2 mM L-glutamine in Neurobasal^®^). On DIV 4–5, we removed half of the growth medium and replaced it with fresh growth medium containing 20 μM 5-fluorodeoxyuridine and 20 μM uridine in order to prevent glial proliferation. Hippocampal neuron cultures were used for the vehicle (control) and 1 μM Aβ, treatment and imaging at DIV 21.

### Organotypic Hippocampal Slice Culture

For the tissue slice studies, we used the C57BL/6J mouse strain. All animal studies were conducted in accordance with National Institutes of Health guidelines and German animal care regulations and approved by the ethical committee on animal care and use of Lower Saxony, Germany.

Hippocampal slice cultures were prepared from 6 to 7 days old mouse pups and processed as described previously (Tackenberg and Brandt, 2009). The brain was separated into two hemispheres and hippocampi were cut out and placed on ice, in a small petri dish with MEM supplemented with 1% glutamine and 1% Pen-Strep. Hippocampi were sliced using a tissue McIllwain chopper (400 μm of thickness) and intact individual slices were selected under microscope and transferred onto membrane inserts with fresh culture medium (MEM, pH 7.2 supplemented with 25% horse serum, 25% BME, 3% glucose, 1% glutamine, 0.5% Pen-Strep and 0.5% fungizone) below the membranes. Tissue cultures were maintained at 37°C and 5% CO_2_ and the culture medium was changed every 2 days. On 11 DIV, culture medium was changed to Neurobasal medium supplemented with 0.5% horse serum (HS), 1% N1, 3% glucose, 1% glutamine, 0.5% Pen-Strep and 0.5% fungizone.

### Preparation of Soluble Oligomeric Aβ_1–42_

**Figure 1:**
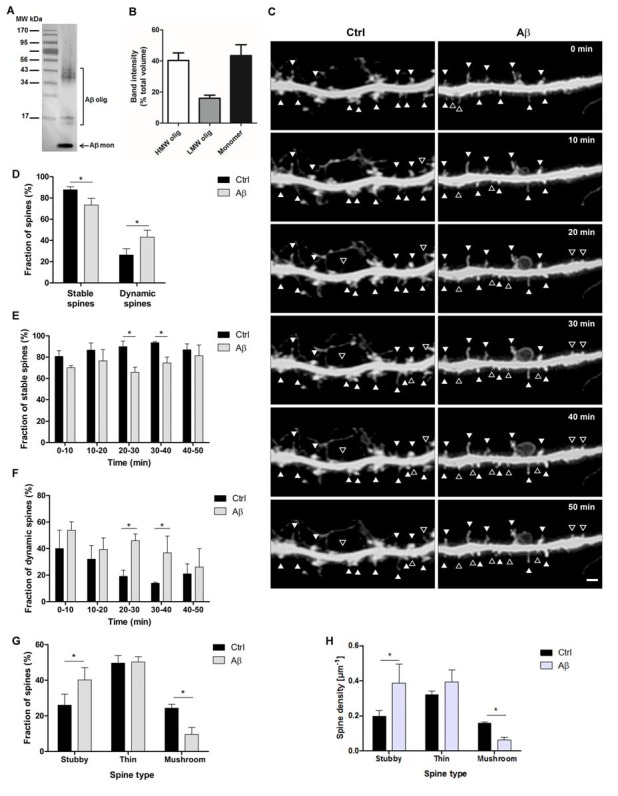
Stability and dynamics of dendritic spine types during short amyloid β (Aβ) treatment. **(A)** Representative western blot shows the broad range of molecular weight protein markers (lane 1) and oligomeric and monomeric forms (lane 2) of Aβ_(1–42)_ preparation probed with monoclonal antibody 6E10. **(B)** The bar graph illustrates the relative quantification (band intensity expressed as the percentage of band volumes of total proteins) of high molecular weight (HMW), low molecular weight (LMW) and monomeric forms of Aβ preparation (*n* = 4 preparations). **(C)** Micrographs show confocal time-lapse images of dendritic segments displaying stable spines (filled arrowheads) and dynamic spines (open arrowheads) at 10-min intervals in control and Aβ conditions. Scale bar, 2 μm.** (D)** Bar diagram representing the cumulative average of dendritic spine stability and dynamics at during 50 min interval. **(E,F)** Bar graphs represent quantification of stability **(E)** and dynamics **(F)** of control and Aβ-treated spines at the indicated 10 min interval. **(G,H)** Detailed analysis of the percentage **(G)** and density **(H)** of the three types of dendritic spines in the 30–40 min time interval. *p < 0.05, compared to non-treated cells; paired one-way ANOVA, *n* = 8 dendrites, of eight neurons (30 spines/dendrite).

Soluble oligomeric Aβ_1–42_ was prepared as reported previously (Dahlgren et al., 2002; Alberdi et al., 2010, 2018). Briefly, Aβ_1–42_ (Bachem, Bubendorf, Switzerland) was initially dissolved to 1 mM in hexafluoroisopropanol (Sigma-Aldrich, Saint Louis, MO, USA) and distributed into aliquots in sterile microcentrifuge tubes. Hexafluoroisopropanol was totally removed under vacuum in a speed vac system and the peptide film was stored dried at −80°C. For the aggregation protocol, the peptide was first resuspended in anhydrous DMSO (Sigma-Aldrich, Saint Louis, MO, USA) to a concentration of 5 mM, to finally bring the peptide to a final concentration of 100 μM in Hams F-12 (PromoCell, Heidelberg, Germany) and to incubate it at 4°C for 24 h. The preparation was then centrifuged at 14,000 *g* for 10 min at 4°C to remove insoluble aggregates and the supernatants containing soluble Aβ_1–42_ were transferred to clean tubes and kept at 4°C. The concentration of Aβ peptides in the soluble phase of the preparation was quantified by absorbance at 280 nm. The measured Aβ_42_ concentration at 100 μM showed a perfect agreement with the calculated concentration based on dilution, as was previously reported (Xue et al., 2017). The contribution of monomers, low molecular and high molecular weight (HMW) oligomers of the Aβ preparation was 40.5 ± 5%, 16 ± 2%, and 43.5 ± 7%, respectively of total Aβ (Figures 1A,B).

### Sindbis Virus Infection

Hippocampal neurons (20 DIV) and organotypic hippocampal slices (12 DIV) were infected with pSinRep5 EGFP virus. Imaging of the dissociated cells was performed after 24 h of EGFP expression (Malinow et al., 2010). In organotypic hippocampal slice culture, virus infection was performed as described previously (Sündermann et al., 2012). Briefly, 12 DIV slices were infected with the pSinRep5 EGFP virus following the droplet method. A low infection rate was performed, which permitted imaging of single neurons at high resolution with minimal overlap of dendritic arbors and allowing imaging of single dendritic fragments. Cultured hippocampal slices were visually examined on 14 DIV to ensure the expression of the fluorescent protein. On 15 DIV, slices were treated with vehicle (control) or 1 μM Aβ or inhibitors and Aβ for 1 h and fixed with cold fixing solution and mounted with the confocal matrix.

### Time-Lapse Imaging of Dendritic Spines and Analysis

Imaging was performed in single labeled spiny hippocampal neurons using a Leica 63× oil objective lens on a Leica TCS SP8 confocal microscopy and a portion of 30 μm of the length of the dendrites was used for quantitative analysis. For time-lapse imaging, stacks were collected every 10 min over a 50-min period to evaluate short-term dynamics. A blind analysis for spine classification, approximate measurements according to previously described criteria was used. Stubby spines have no constriction between the head and the dendritic shaft. Thin spines have a small head and a neck of similar magnitudes. Mushroom spines have large heads and narrow necks.

Several parameters were measured to obtain detailed information of the dynamics of the three types of spines at each observation interval as previously described (Huang et al., 2015): the number of spines added and eliminated, and the number of spines maintained from one observation to the next. The maintained spines were classified as stable, whereas the added and eliminated spines were considered dynamically changed. Spine stability was defined as the percentage of stable spines compared with previously observed ones. Spine addition was defined as the percentage of added spines compared with the next observed ones. Spine elimination was defined as the percentage of eliminated spines compared with previously observed ones. From the starting imaging point to the next, an eliminated spine may disappear directly, or transform into other spine types. Therefore, the percentages of spines that appeared or disappeared, as well as those of each spine type that transformed into the other types, were calculated. Data are presented as mean ± standard error of the mean (SEM) of a cumulative average of each 50 min observation interval or each 10 min interval observation.

### Hippocampal Slices Imaging and Analysis

A blind analysis of dendritic spine density was performed using Nikon cLSM Eclipse TE 2000U equipped with 60× objective (NA: 1.4) and 488 nm Argon laser. Dendritic branches of individual CA1 pyramidal neurons on the apical side were imaged with a voxel size of 0.08 × 0.08 × 0.20 μm in the x-y-z directions. Images were deconvoluted with Autodeblur 9.3 software and spine analysis was performed with 3DMA Version 0204 software (Tackenberg and Brandt, 2009).

Confocal high-resolution microscopy of complete CA1 hippocampal neurons was performed as described previously (Golovyashkina et al., 2015). Each single neuron was imaged with Zeiss 510 META in 8–12 overlapping image stacks with voxel size 0.30 × 0.30 × 0.45 in x-y-z directions. 3D reconstruction of whole neurons was performed using Neuromantic software (University of Reading, Reading, UK) in semiautomated mode. Sholl analysis was performed separately for basal and apical parts of dendritic trees.

### Statistical Analysis

All data were expressed as mean ± SEM. Statistical analyses were performed using absolute values. GraphPad Prism software was used applying one-way or two-way analysis of variance (ANOVA) with the *post hoc* Fisher’s least significant difference (LSD) test for multiple comparisons. Comparisons between two groups were analyzed using the paired student’s one-tailed *t*-test.

## Results

### Short-Term Spine Stability and Dynamics Is Modulated by Aβ Oligomers in Cultured Hippocampal Neurons

To explore the effect of acute treatment with soluble Aβ_42_ (Figures 1A,B) on stability and dynamics of dendritic spines in living cells, hippocampal neurons (21 DIV) were infected with EGFP Sindbis virus to monitor spine morphology by confocal microscopy on dendrites of control and 1 μM Aβ-treated neurons (Figure 1C). Images of dendrites were acquired at 10 min intervals for 1 h. Stable and dynamic spines were counted every 10 min, and the combined average of each time interval was represented (Figure 1D). We observed that although many spines remained stable in control and Aβ-treated cells, there was a significant decrease in the proportion of stable spines of Aβ-treated samples when compared to control (73.4 ± 6.2% and 87.7 ± 2.8%, respectively; *p* < 0.05). Accordingly, a higher number of spines were significantly added, eliminated or modified in the Aβ-treated dendrites compared with controls (43.1 ± 6% and 26.3 ± 6%, respectively, *p* < 0.05). Neither the added nor the eliminated spines showed any significant difference when analyzed separately. Detailed analysis at each observed time point showed that stability of spines on the Aβ-treated dendrites was significantly lower and the dynamics were higher than that in control dendrites. Significant differences were observed during the 20–30 min and 30–40 min periods (*p* < 0.05; Figures 1E,F). These results suggest that dynamic changes mostly occur between 20 and 40 min of the whole-cell recording.

Next, in order to specifically study dynamics of the different spine types with Aβ-treatment, neuronal protrusions were classified as thin, stubby or mushroom spines based on their morphology, and each spine type was counted in the 30–40 min period. Stubby and mushroom spines were more dynamic than the thin spines during Aβ-treatment since the percentage and density of stubby spines were higher and mushroom spines were lower after Aβ-treatment compared to control (Figures 1G,H). A number of thin spines remained unchanged between both conditions. Overall, these results suggest that Aβ oligomers induce dynamics of stubby and mushroom spines in a short period of time.

### Aβ-Oligomers Promote an Increase in Spine Density Through CaMKII and Integrin β1 in *ex vivo* Hippocampal Cultures

**Figure 2:**
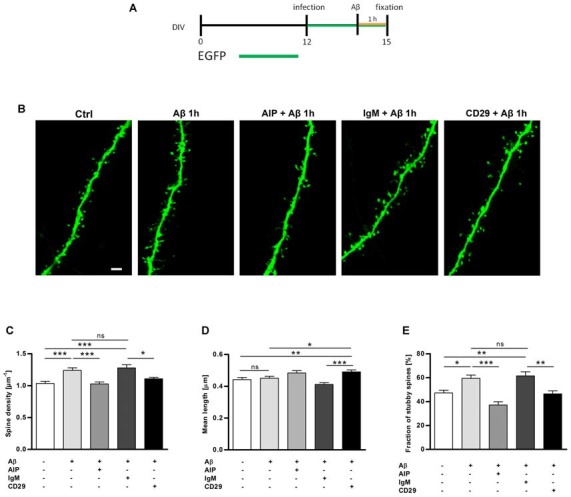
Hippocampal slices treated with Aβ oligomers show an increase in the total dendritic spine density, which is reverted with CaMKII and integrin β1 inhibitors.** (A)** Organotypic hippocampal slices were infected with sindbis virus expressing EGFP at day 12 *in vitro*. Three days later, slices were treated with Aβ oligomers 1 μM or vehicle and fixed at day 15 *in vitro* as indicated on the timeline. **(B)** Micrographs obtained by confocal imaging show apical dendritic segments from CA1 hippocampal neurons in control and Aβ treatment conditions in the presence of different inhibitors, AIP (CaMKII inhibitor) and CD29 (integrin β1 inhibitor). Scale bar, 2 μm. **(C,D)** Bar graphs represent quantification of spine density and spine mean length in different cultures. **(E)** The bar graph shows quantification of stubby spine density after Aβ treatment in the presence or absence of inhibitors. *p < 0.05, **p < 0.01, ***p < 0.001, n.s. non-significant, compared to non-treated cells; paired one-way ANOVA. Data are represented as mean ± standard error of the mean (SEM), *n* = 35 dendrites of 5–6 neurons per condition.

Next, to determine the functional pathway of Aβ oligomers that induce spine changes in an authentic central nervous system (CNS) environment, organotypic hippocampal slices from mice were infected with the neurotropic Sindbis virus expressing EGFP at day 12 *in vitro*. Three days later, slices were treated with 1 μM Aβ oligomers for 1 h and immediately fixed (Figure 2A). Efficient infection of neurons in all regions of hippocampal slices was observed at 3 days post-infection by the intense EGFP fluorescence at higher magnification, similar to the previous study (Tackenberg and Brandt, 2009). Single CA1 neurons were identified according to their morphology and could be imaged by confocal high-resolution imaging and individual spines of dendritic segments were evaluated by 3DMA analysis and by an algorithm-based 3D reconstruction, respectively (Tackenberg and Brandt, 2009; Golovyashkina et al., 2015).

To determine changes regarding spine density, dendritic segments of *stratum radiatum* of EGFP-expressing CA1 neurons were imaged (Figure 2B). Quantitative analysis of dendrites treated with Aβ oligomers clearly showed a significant increase in spine density compared to untreated neurons (Figures 2B,C; 1.24 ± 0.04 μm^−1^ vs. 1.03 ± 0.03 μm^−1^, ***p < 0.001). However, no significant differences were observed in the mean length of the spines (Figure 2D; 0.451 ± 0.11 μm vs. 0.442 ± 0.12 μm).

Aβ oligomers require the activity of protein kinase CaMKII and receptor integrin β1 to induce synaptotoxicity and astrogliosis, respectively (Wyssenbach et al., 2016; Opazo et al., 2018). To determine whether CaMKII and integrin β1 were also involved in acute Aβ-mediated spine modifications, EGFP-expressing slices were pretreated with the CaMKII inhibitor AIP, the integrin β1 blocker antibody CD29, and the isotype control antibody IgM (Yokosaki et al., 1994; Ishida et al., 1995). We observed that AIP inhibitor and CD29 antibody abolished the increase in spine density induced by Aβ and Aβ + IgM, respectively on CA1 hippocampal neurons (Figures 2B,C; 1.03 ± 0.03 μm^−1^ vs. 1.24 ± 0.04 μm^−1^ and 1.11 ± 0.02 μm^−1^ vs. 1.28 ± 0.05 μm^−1^, ***p < 0.001). Overall, these results show that a short term treatment of Aβ oligomers increases significantly the spine density of CA1 neurons in organotypic hippocampal slices. Spine density increment induced by Aβ oligomers was prevented by AIP and CD29 inhibitors, confirming that mechanistically CaMKII and integrin β1 participated as early modulators of spine dynamics.

**Figure 3:**
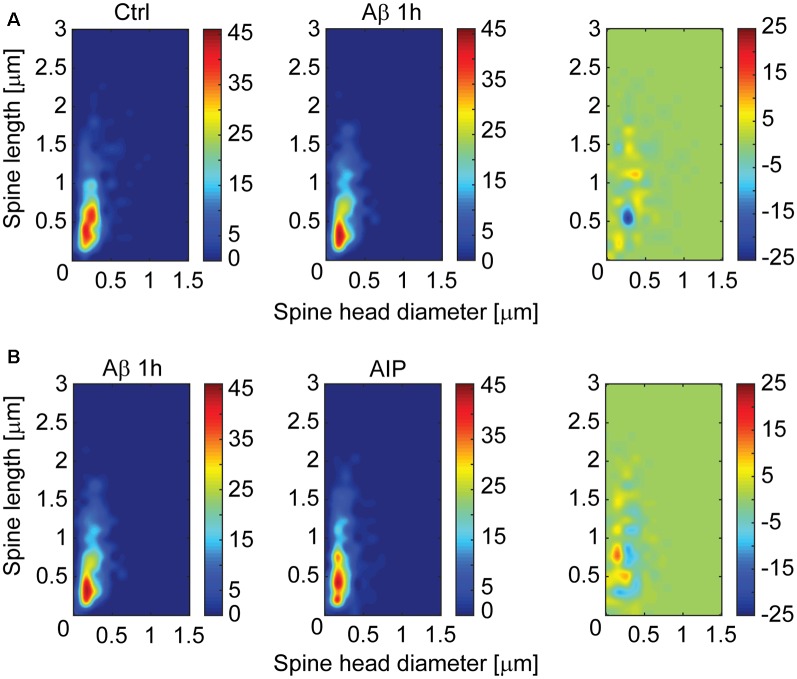
CaMKII inhibitor shifts Aβ-induced spine morphology alterations on hippocampal CA1 neurons as shown by a multidimensional spine density analysis. Multidimensional spine density histograms representing equal numbers (600 random dendritic spines/condition) of spines from control and Aβ treated samples **(A)** or Aβ treated samples in presence or absence of CaMKII inhibitor **(B)**. Heatmap graphs were derived by plotting spine densities as a function of spine length × head diameter for each sample. Difference plots were derived by comparing group Aβ values vs. control **(A, right)** or CaMKII + Aβ values vs. Aβ alone values **(B, right)**. Note that the presence of CaMKII inhibitor shifts spine parameters back towards lower head diameter.

For further characterization of spine changes, spines were classified as mushroom, stubby and thin (Peters and Kaiserman-Abramof, 1970), by using an algorithm-based computer-assisted method (Tackenberg and Brandt, 2009). In the CA1 region, the fraction of stubby spines significantly increased after Aβ treatment when compared with control (Figure 2E; 59.4 ± 2.7% vs. 47.2 ± 2.3%, respectively; *p* < 0.001). Interestingly, inhibitors of CaMKII and integrin β1, AIP and CD29 antibody respectively, strongly reduced the Aβ-induced increment of stubby spines (Figure 2E; 37.12 ± 2.7% for AIP+Aβ treatment; *p* < 0.001; and 46.4 ± 2.5% vs. 61.5 ± 3.5% for CD29+Aβ vs. IgM+Aβ; *p* < 0.05). We did not observe any significant influence of Aβ oligomers on the fraction of thin and mushroom spines as compared with control treatments (32.5 ± 3% vs. 26.6 ± 1.9% and 32 ± 1.7% vs. 29.7 ± 2.3%, respectively). Using a distribution based depiction of the differences with treatment that is independent of any classification of the spine types, we observed that inhibition of CaMKII activity induced a strong shift back towards spines with smaller heads after spine parameter alterations induced by Aβ oligomers (Figures 3A,B).

Altogether, these results suggest that an acute Aβ treatment produces an increase in stubby spine density in CA1 hippocampal neurons, which is mediated by both CaMKII and integrin β1 activity in an *ex vivo* model of AD using organotypic hippocampal slice cultures.

### Aβ Oligomers Induce Early Morphological Changes Promoting Dendritic Complexity in CA1 Hippocampal Neurons

**Figure 4:**
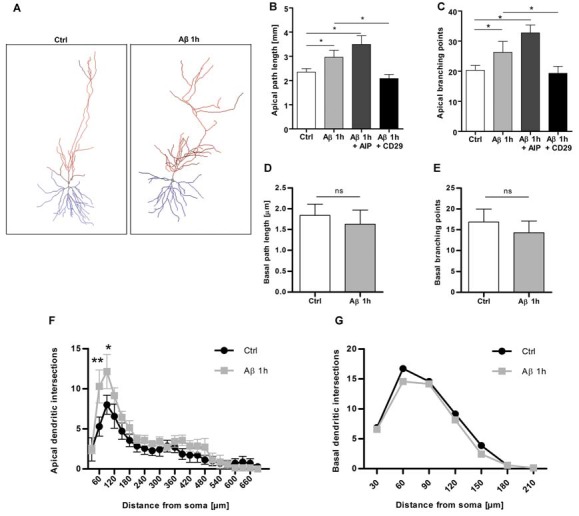
Aβ oligomers induce dendritic complexity at sites of Schaffer collateral input. **(A)** 3D reconstructions of representative CA1 neurons in untreated and Aβ-treated cells. **(B–E)** Quantitative analysis of dendritic complexity as determined from apical path length** (B)**, apical branching points **(C)**, basal path length **(D)** and basal branching points **(E)**. **(F,G)** Sholl analysis of the dendritic tree in CA1 neurons after Aβ treatment indicates higher branching in the proximal part of the apical but not in basal dendrites. *p < 0.05, **p < 0.01, n.s. non-significant, compared to non-treated cells; paired student’s *t*-test **(B–E)** or two-way ANOVA **(F,G)**. Data are represented as mean ± SEM, *n* = 7 neurons per condition.

The chronic presence of Aβ peptides induces region-specific dendritic simplification in an *ex vivo* model of AD (Golovyashkina et al., 2015). To investigate the early events on alterations in dendritic arborization induced by Aβ oligomers, organotypic hippocampal slice cultures were infected with Sindbis virus mediating EGFP expression and cultures were treated with 1 μM Aβ oligomers for 1 h. In order to obtain a quantitative assessment in total path length and number of branching points, we used an algorithm-based 3D reconstruction of whole CA1 neuron morphology. As shown in (Figure 4A), Aβ oligomers increased dendritic complexity in apical CA1 neurons compared to untreated cells. Quantitative analysis of dendritic complexity in presence or absence of Aβ oligomers clearly showed a significant increase in both total path length (Figure 4B; from 2.35 mm ± 0.14 to 2.96 mm ± 0.29, *p* < 0.05) and number of branching points (Figure 4C; from 21.33 ± 1.4 mm to 28.3 ± 3.5 mm) of apical neurons. However, no changes were observed in total path length or number of branching points in the basal part of CA1 neurons (Figures 4D,E). Interestingly, significant differences in the dendritic morphology were observed between Aβ-treated and CD29+Aβ-treated cultures but not with AIP+Aβ cells (Figures 4B,C), thus suggesting the involvement of integrin β1, but not of CaMKII, as an intermediary of Aβ signaling.

Furthermore, since CA1 pyramidal neurons receive different inputs in specific subregions of the dendritic tree, we examined subregional differences in the induction of dendritic complexity mediated by Aβ. For that, 3D Sholl analysis was performed on basal and apical branches to quantify changes in the intersection frequency of dendrites as a function of the distance from the cell body. We observed that, in the apical part, dendritic complexity was induced specifically in one segment of the tree which was localized at 15–25% of the total apical length, correlating with regions where most inputs from Schaffer collateral occur (Figure 4F). In contrast, no changes in dendritic intersections localized in the basal part were found (Figure 4G).

Therefore, analysis of dendritic complexity based on a 3D reconstruction of the whole CA1 neuron morphology indicates an increase in the apical dendrite length and branching points, at sites of Schaffer collateral input as an early event of Aβ oligomer-induced dendritic changes.

## Discussion

Loss of dendritic spines and changes in dendritic morphology are early pathological features characterizing AD (Bakota and Brandt, 2016). To date, however, the molecular events triggered by the acute presence of soluble Aβ oligomers in spine dynamics and dendritic changes are not fully understood. Our findings indicate that Aβ oligomers acutely modulate the structural plasticity of spines in primary cultures of rat and murine hippocampal neurons. Moreover, a detailed spine analysis of dendritic segments of CA1 pyramidal neurons confirmed that the number of total spine density, with the stubby spines as a major contributor, was significantly increased by Aβ. Surprisingly, Aβ oligomers also promoted a higher complexity in apical CA1 dendrites at sites of Schaffer collateral input in CA1 neurons. Mechanistically, we showed that blockade of integrin β1 abolished the spine and dendritic changes but CaMKII inhibition only reduced the spine changes. Altogether, these data revealed that Aβ oligomers exert a positive effect on synapses in selected areas of the hippocampus. Our findings could be understood as early compensatory mechanisms prior to the synaptic damage described in previous works.

Previous studies showed that increased levels of Aβ in a soluble, oligomeric form causes spine changes (Shankar et al., 2007; Tackenberg and Brandt, 2009; Tackenberg et al., 2009; Penazzi et al., 2016). Thus, chronic exposure to Aβ in organotypic hippocampal slice cultures from transgenic mice expressing mutated APP (APP_SDL_) showed that the density of spines was reduced in most regions. Additionally, the spine shapes progressively changed from mushroom to stubby with the time of Aβ exposure in an NMDA receptor-dependent pathway (Tackenberg and Brandt, 2009), but no selective loss of mushroom spines was found (Penazzi et al., 2016). In agreement with these results, physiological concentrations of naturally secreted Aβ oligomers, but not monomers, induced progressive loss of hippocampal synapses (Shankar et al., 2007). In contrast, short exposure of the peptide (in the range of minutes) enhanced synaptic plasticity while longer exposures lasting several hours had deleterious effects on it (Koppensteiner et al., 2016). Together, these reports support a model for the onset of AD in which synaptic dysfunction and memory loss are triggered by prolonged exposure of oligomeric Aβ. Here, we analyzed spine dynamics, i.e., change in spine shapes and the appearance or loss of spines over time in living cell cultures. Accordingly, we observed that soluble forms of Aβ peptide or Aβ oligomers, as the active components (Alberdi et al., 2018), modulated spine dynamics within a very short-term time window. Overall, most dendritic spines were highly stable under basal conditions, whereas after treatment with Aβ oligomer stubby and mushroom spines showed the most dynamic the most dynamic behavior. These results suggested that both spine types were more susceptible to rapid morphological changes induced by Aβ than thin spines in cultured hippocampal neurons. However, during culture preparation, synaptic contacts are disrupted and have to be reformed *in vitro*. To avoid this feature, we used hippocampal slice cultures, where connections remain largely intact. In this authentic CNS environment, an acute Aβ treatment increased the fraction of stubby spines but no changes were observed regarding mushroom type. Altogether, these results suggest that spines become remarkably dynamic when Aβ oligomers interact with dendrites, rapidly generating excess and transitory synaptic connections. Further evidence will be needed to identify whether these findings are compensatory mechanisms observed in the early stages of AD (Bobkova and Vorobyov, 2015).

A large number of possible Aβ receptors have been suggested (Jarosz-Griffiths et al., 2016). Interaction between integrins, a large family of extracellular matrix receptors, and Aβ peptides promote neurotoxicity and inhibition of LTP (Wang et al., 2008), mitochondrial and synaptic dysfunction (Woo et al., 2015), gliosis (Wyssenbach et al., 2016) and oligodendrocyte differentiation Quintela-López et al., 2019). In the developing and adult brain, many integrins are present at high levels at synapses (for review, Park and Goda, 2016). Functional regulation of neuronal structure has been described for integrin β1. Selective loss of this receptor in excitatory neurons leads to reductions in the size and complexity of hippocampal dendritic arbors, hippocampal synapse loss and impaired hippocampus-dependent learning (Warren et al., 2012). Additionally, integrin β1 mRNA and protein levels are upregulated by Aβ oligomers in astrocytes and oligodendrocytes suggesting that Aβ peptides might change the dynamic nature of integrin expression and function throughout AD (Wyssenbach et al., 2016; Quintela-López et al., 2019). Notably, we observed that spine density and specifically the fraction of stubby spines returned to control levels when integrin β1 was reduced with blocking antibodies. These results suggest that integrin β1 participates in the regulation of spine changes by Aβ oligomers. A key actin-binding protein in the calcium signaling cascade is Ca^2+^/calmodulin-dependent kinase II (CaMKII; for review, Zalcman et al., 2018). The high abundance of CaMKII in dendritic synapses suggests that kinase might participate in establishing the structure of the cytoskeleton in the dendritic spine. In fact, CaMKII activating stimuli trigger dissociation of CaMKII form F-actin bundles and, during a brief time interval, the actin cytoskeleton can be remodeled in the dendritic spines (Kim et al., 2015). Our data suggest that Aβ oligomers, integrin β1 and CaMKII signaling governs spine changes in hippocampal neurons.

Interestingly, integrin β1 but not CaMKII inhibition abolished the Aβ-mediated rapid reorganization of the dendritic branch, which led to increased apical dendritic arbor. The development and rearrangement of dendrites and dendritic spines are timely, very specifically and distinctly regulated (Koleske, 2013) therefore, it is highly likely that it also requires the involvement of different mechanistic procedure. This is further confirmed by observations that in various psychiatric and neurodegenerative disorders the patterns of dendritic spine and dendrite branch loss differ from each other (Kulkarni and Firestein, 2012).

In summary, we provide evidence that Aβ may promote dual effects by acutely enhancing dendritic complexity and spine density. Further evidence will be needed to identify whether these mechanisms precede synaptic damage observed in more advanced stages of the disease.

## Data Availability Statement

The raw data supporting the conclusions of this article will be made available by the authors, without undue reservation, to any qualified researcher.

## Ethics Statement

The animal study was reviewed and approved by Ethical committee on animal care and use (CEEA) of UPV/EHU, Spain, and ethical committee on animal care and use of Lower Saxony, Germany.

## Author Contributions

CO-S designed, performed and analyzed experiments. CO-S wrote the article. AG-B and JV contributed to data analysis. LB and RB designed experiments, contributed to data analysis and discussion and to manuscript review. JZ, CM, and EA designed and analyzed experiments and wrote the article.

## Conflict of Interest

The authors declare that the research was conducted in the absence of any commercial or financial relationships that could be construed as a potential conflict of interest.

The handling Editor declared a shared affiliation, though no other collaboration, with one of the authors CM.
